# Effectiveness, Tolerability, and Acceptance of a Food Supplement in the Management of Non‐Illness‐Related Hair Loss and Thinning

**DOI:** 10.1111/jocd.70852

**Published:** 2026-05-17

**Authors:** Ehrhardt Proksch, Sandra Bartylla

**Affiliations:** ^1^ Clinic for Dermatology, Allergology and Venereology, University Hospital Schleswig‐Holstein, Campus Kiel Kiel Germany; ^2^ Steigerwald Arzneimittelwerk GmbH Darmstadt Germany

**Keywords:** anagen, biotin, diffuse hair loss, L‐cystine, millet extract, pantothenic acid, thinning hair

## Abstract

**Background:**

Diffuse hair loss and shedding is often managed with food supplements, yet evidence for ingredient combinations is limited. Our exploratory study evaluated a nutritional supplement (Priorin capsules, Bayer) containing millet extract, pantothenic acid (vitamin B5), L‐cystine, and biotin (vitamin B7) in healthy subjects with light diffuse hair loss.

**Methods:**

In this open‐label, intra‐individual, multicenter study, subjects with non‐illness‐related, light diffuse hair loss used the product (two capsules/day) for 12 weeks. Endpoints included objective measurements of hair density, hair growth coefficient, and hair loss (phototrichogram), subjective evaluations of hair appearance, beauty and volume (hairdresser‐rated), consumer perception and acceptance, and tolerability.

**Results:**

In 112 subjects (mean age 32 ± 1 years; 93.8% female), hair density (*n* = 109) and the proportion of hairs in the anagen phase increased significantly, while the telogen proportion decreased (*p* < 0.001, week 12 vs. baseline). Hair loss had declined by 59% at week 12 (*p* < 0.001). Significant improvements in the hairdresser‐rated hair quality and appearance parameters (shine, softness/elasticity, strength, smoothness, dryness, vitality, volume) were observed (*p* < 0.001). From week 2, subjects reported progressive improvement in appearance, growth, and slowing of hair loss. At week 12, 90% of subjects perceived improved hair appearance and growth, 85% reported slowing down of hair loss, and 82% were satisfied with the overall hair improvements with treatment. The supplement was generally well tolerated.

**Conclusions:**

The combination of millet extract, pantothenic acid, L‐cystine, and biotin was associated with reduced hair loss, improvements in hair growth and appearance, and high subject satisfaction, and was well tolerated over 12 weeks.

## 1 Introduction

Diffuse hair loss is a common but under‐recognized condition that affects the whole scalp. The generalized hair loss and overall thinning of hair that characterizes the disorder [1] can substantially affect quality of life [2]. Both sexes can be affected [3]; however, women may suffer more because dense hair is often perceived as important for feminine beauty, in contrast to men in whom hair loss is undesirable but widely accepted. Diffuse hair loss can begin at around 30 years of age in women and increases with age [4].

**FIGURE 1:**
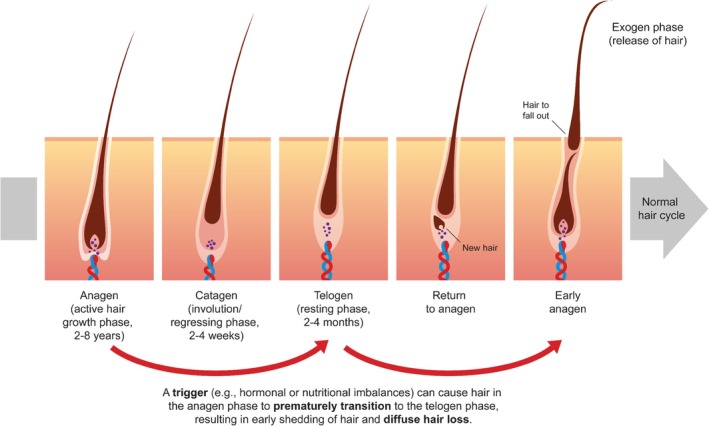
The normal hair cycle can be disrupted by triggers that cause diffuse hair loss.

There are several causes of diffuse hair loss, the most common being telogen effluvium (where anagen‐phase hair follicles prematurely transition to the telogen phase, resulting in increased hair shedding) [5]. The disruption in the hair cycle (Figure 1) can be triggered by a variety of factors, including prolonged physical or emotional stress, significant hormonal changes (e.g., taking or stopping hormonal contraceptives, pregnancy and especially after childbirth, being postmenopausal), acute and chronic infections, seasonal factors, and significant nutritional deficiencies, such as those caused by restrictive diets [3, 4, 6]. In parallel, mechanical factors such as the heat from straighteners or curling irons may exacerbate poor hair structure, hair breakage, and subsequent thinning hair. Hair loss may be temporary (like after childbirth or infections) or progressive (like in aging). Even after temporary hair loss, full recovery of the previous hair density sometimes does not occur.

Although there are minimal harmful physical effects, diffuse thinning can be psychologically damaging, associated with impaired self‐esteem, reduced body image satisfaction, and higher rates of anxiety and depression—particularly among women, for whom hair often represents an important aspect of identity and social appearance [2, 7, 8]. Diffuse hair thinning is usually temporary and reversible—yet the psychosocial burden is such that many affected individuals actively seek therapeutic options. However, management can be complex because multiple triggers can be involved [3]. Those affected often try many different products in an attempt to maintain and improve their hair appearance, from cosmetics such as shampoos, leave‐on products, and hair masks to food supplements.

Against this background, clinical trials assessing novel or supportive treatments for diffuse hair loss are warranted to address an area of high unmet need. In particular, many food supplements for hair loss are not supported by data, especially when various ingredients are used in combination. Therefore, we carried out an exploratory study to investigate the impact of a food supplement (Priorin capsules, Bayer) used in people with thinning hair for the maintenance of strong, healthy hair. Priorin is marketed in approximately 30 countries and is a well‐known brand for the management of hair loss, thinning hair, and for overall hair beauty and appearance. The product combines millet extract, pantothenic acid (vitamin B5), L‐cystine, and biotin (vitamin B7)—ingredients with a well‐established role in hair health. Millet seeds contain miliacin, which stimulates proliferation of keratinocytes and cell proliferation in the hair bulb, prolongs the anagen phase, and reduces the telogen phase [9, 10]. Pantothenic acid is essential for energy production, hormone synthesis and activation, and other processes critical for hair growth [11, 12, 13]. Biotin is recommended to support hair growth, acting as a coenzyme in metabolic processes and supporting the hair texture [3, 14]. L‐cystine is a key building block of keratin [15]; when used in combination with thiamine, calcium pantothenate, and folic acid, it contributes toward enhanced metabolic activity, cell viability, and keratinocyte proliferation while protecting against oxidative stress [16]. In vitro analysis showed that the combination of millet extract, L‐cystine, and pantothenic acid increased keratinocyte proliferation and metabolic capacity to a greater extent than when the ingredients were evaluated alone, suggesting a beneficial effect on keratinocyte growth and stimulation when using the combination [17]. Furthermore, the combination had modulatory effects in human hair follicles in vitro, including an enhanced rate of anagen hair, keratinocyte proliferation, and stimulation of the growth factor IGF‐1 [18].

Thus, our rationale was that when used in combination, these ingredients could increase hair density and reduce hair loss. We also evaluated the hairdresser‐rated efficacy of the product on hair appearance, volume, and beauty, consumer acceptance in terms of satisfaction with hair appearance and growth, and tolerability. Our findings will add to the evidence base for the combined use of food supplements in diffuse hair loss and help people to make informed choices in the management of their condition.

## 2 Materials and Methods

### 2.1 Study Design

This was an open‐label, intra‐individual, multicenter study in healthy subjects, performed at two sites in France and Poland. As the main objective was to evaluate the product efficacy in terms of hair loss and hair density, subjects with non‐illness‐related, light diffuse hair loss and thinning of hair were included.

Our exploratory food study did not require Ethics Committee Approval or the Competent Authority Authorization from these countries. The study adhered to the Declaration of Helsinki and local French and Polish regulations. All subject data collected were anonymous and confidential and collected in electronic files that were submitted for authorization to the Commission Nationale de l'Informatique et des Libertés.

### 2.2 Inclusion and Exclusion Criteria

The full list of inclusion and exclusion criteria is provided in Table S1. Eligible participants were healthy Caucasian men and women aged 18–45 years with Fitzpatrick skin type I–IV and a history of mild seasonal hair loss. Subjects were required to provide written informed consent before any study‐specific procedures were performed, agree to protocol adherence, and (for women of childbearing potential) have a negative pregnancy test and reliable contraception. Key hair‐related requirements included < 15% hair in the telogen phase in women (< 20% in men) a maximum 8 weeks before inclusion (i.e., no chronic hair loss), evidence of *increased* telogen proportion at inclusion (to ensure the seasonality of the diffuse hair loss), weakened and devitalized hair (e.g., hair dryness, reduced hair volume), hair of ≥ 6 cm length, and willingness to maintain this length and allow a 1 cm^2^ scalp area to be shaved for phototrichogram analysis. Hair needed to be brown or of chestnut color that was easily visible on photographs.

Major exclusion criteria included scalp disorders (e.g., seborrheic dermatitis, alopecia, eczema, psoriasis), systemic diseases or medications affecting hair growth/loss within 6 months, allergies to the components of the food supplement, pregnancy or breastfeeding, and concurrent participation in another clinical or cosmetic trial.

In addition, hair dying was not allowed within 3 weeks prior to baseline or during the study, the hairstyle at baseline had to be maintained throughout the study, and medical and cosmetic products for treating hair loss, hair beauty products, or products against dermatological diseases of the scalp (e.g., antidandruff products) were prohibited 30 days before baseline and throughout the study. All subjects were recruited between Sep 5, 2016, and Oct 17, 2016.

### 2.3 Investigational Product

The product investigated (Priorin capsules, provided by the study sponsor, Bayer Consumer Care) was capsules for oral administration that contained millet extract (210 mg), pantothenic acid (vitamin B5; 14 mg), L‐cystine (3 mg), and biotin (vitamin B7; 100 μg), along with excipients. Two capsules were taken every day for 12 weeks—one in the morning, one in the evening. Each capsule was swallowed with a glass of water (150 mL).

### 2.4 Endpoints

Baseline demographic characteristics, medical history, prior and concomitant therapies were collected. Treatment compliance and illustrative photographs were also recorded. Outcomes included the following, with further details in Table S2:

- **Hair growth parameters** (hair density, proportion and density of anagen and telogen hairs, and hair growth coefficient [anagen/telogen ratio]), assessed by phototrichogram at baseline and after 4, 8, and 12 weeks.
- **Hair loss**, measured by standardized hair washing with collection and counting of shed hairs at baseline and after 4, 8, and 12 weeks.
- **Hair appearance** (shine, softness, strength, smoothness, thickness, dryness, vitality, and volume), rated by a professional hairdresser on a 10‐point scale at baseline and after 4, 8, and 12 weeks. Higher scores indicated better hair quality.
- **Consumer acceptance**, assessed weekly by subject questionnaires covering satisfaction with hair appearance, hair growth, slowing down of hair loss, frontal hairline, and overall hair status.
- **Safety outcomes**, including clinical examination and adverse event (AE) reporting at each visit, tolerability assessment by a physician, and documentation of withdrawals due to AEs.

Subjects attended visits at baseline and after 4, 8, and 12 weeks of using the product (Table S3). At each visit, efficacy assessments included phototrichogram analysis, standardized hair wash with hair count, and professional hairdresser evaluation of hair appearance, beauty, and volume. Subject questionnaires were completed weekly, and clinical safety evaluations with AE monitoring were performed throughout the study.

### 2.5 Statistical Analysis

As this was an exploratory study, no formal sample size calculation was performed. We planned for 150 subjects to be selected and 120 to be included to account for screening failures and dropouts and achieve our objective of 100 evaluable subjects. This number was standard for this kind of study and was considered sufficient to reach the study objectives based on previous experiences.

The analysis populations included the full analysis set (FAS; any subject who received at least one dose of the study intervention and had at least one patient‐reported outcome assessment available for the specific endpoint), which was used for the efficacy endpoints; the per‐protocol population (PP; any subject who used the food supplement at least once, without any major protocol deviation), used for sensitivity analysis; and the safety population (any subject who used the food supplement at least once).

Each quantitative variable was summarized by time point using descriptive statistics (mean ± standard deviation). Qualitative data were summarized by frequency (n/N; number of observations for one variable/total number of observations) and percentage (%). For each quantitative variable, a mixed effect model (PROC MIXED) for repeated measures was fitted to raw data including the factors: time as fixed (weeks 0, 4, 8, and 12), subject as random. All statistical tests were assessed by *α* = 5% two‐sided level of significance vs. baseline in a bilateral approach. No strategy for missing data was defined. Non‐valid data were considered and treated like missing data.

## 3 Results

**FIGURE 2:**
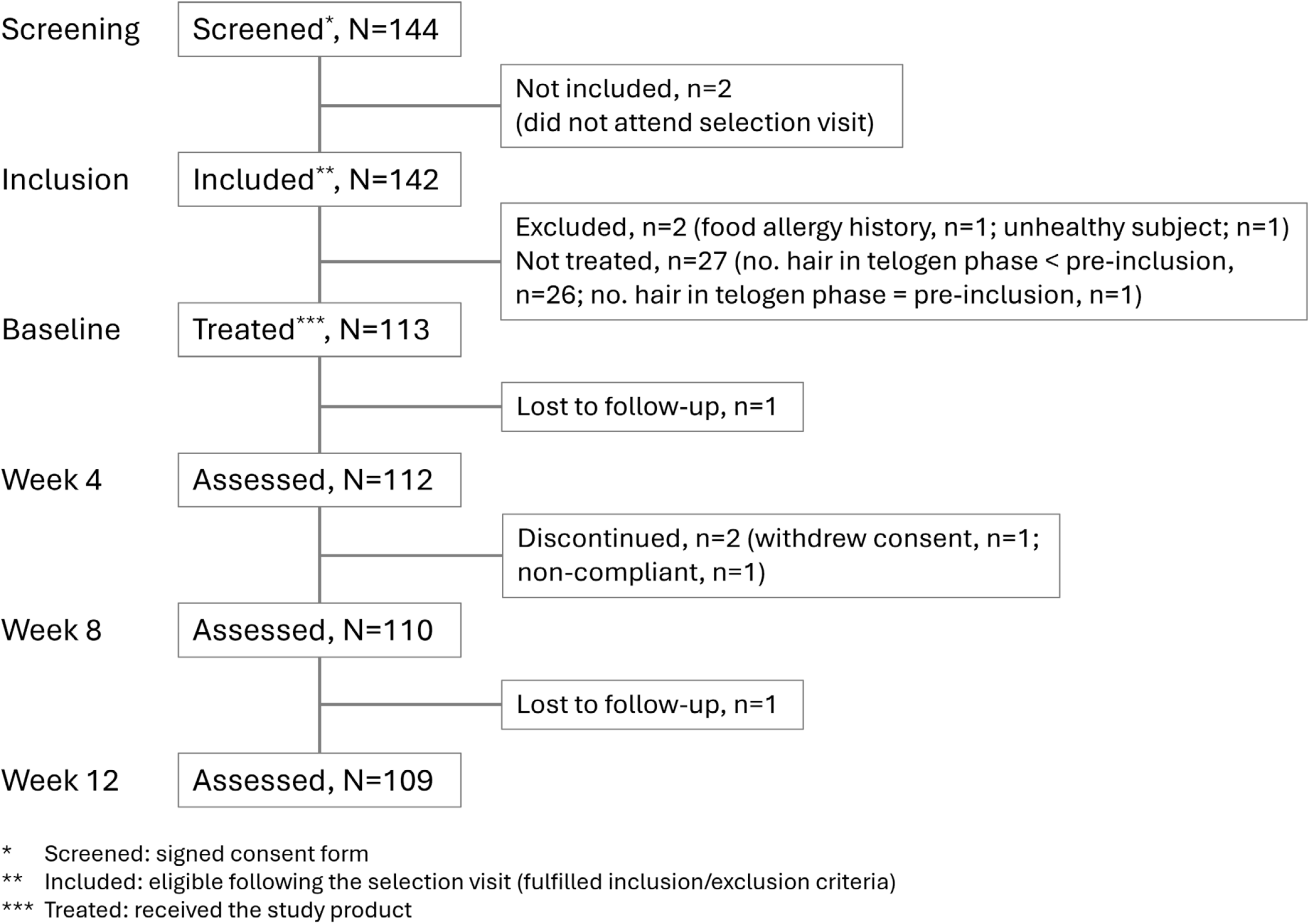
Subject disposition from baseline to week 12.

The study took place over four months (Sep 5, 2016, to Jan 11, 2017). Initially, 144 subjects signed the consent form and were screened (Figure 2). Two were not included as they missed the selection visit. Thus, 142 subjects fulfilled the inclusion and exclusion criteria and were included in the study. Of these, 113 received the study protocol and the investigational product. Four subjects did not complete the study (two subjects were lost to follow‐up, one withdraw informed consent, one discontinued because of a protocol deviation (serious non‐compliance)). Therefore, 109 subjects completed the study (54 in France, 55 in Poland). The study duration for each subject was 86 days (i.e., treated and followed‐up between Nov 30, 2016, and Jan 11, 2017). The FAS population consisted of 112 subjects (one subject had been wrongly included at baseline after a change in oral contraception), who also formed the safety population. The PP population consisted of 102 subjects (11 were excluded for protocol violations).

The mean age of the FAS population was 32 ± 1 years and 105 (93.8%) were female. Subjects were generally healthy; the most frequent baseline medical histories were headache/migraine and mild allergies. No serious concomitant conditions were reported. Concomitant medications were common, occurring in 74 (66.1%) subjects, but no relevant drug interactions were expected. At 12 weeks, the compliance rate was 100% on average.

### 3.1 Hair Growth (Phototrichogram)

**FIGURE 3:**
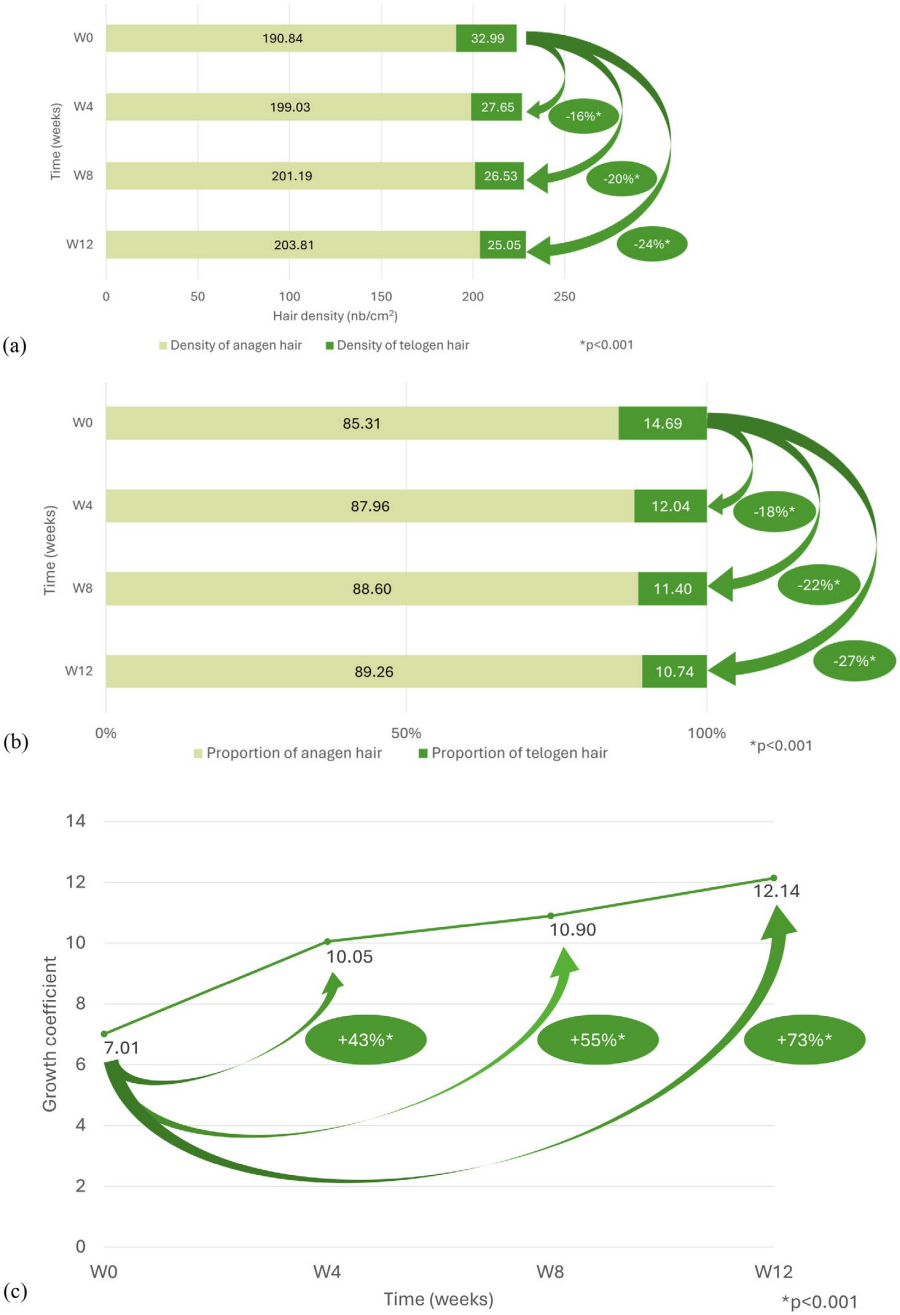
Change in hair growth from baseline to week 12: (a) total hair density (hair/cm^3^); (b) proportion (%) of hair phase; (c) hair growth coefficient (anagen/telogen).

All results for hair growth parameters are listed in Table S4. Over the 12‐week treatment period, total hair density had increased at weeks 8 (*p* = 0.005) and 12 (*p* < 0.001) compared with baseline (Figure 3a). At weeks 4, 8, and 12, there was a significant increase in the proportion of hairs in the anagen phase (3%, 4%, and 5%, respectively; *p* < 0.001) and a significant decrease in the proportion in the telogen phase (18%, 22%, and 27%; *p* < 0.001) (Figure 3b). The hair growth coefficient (anagen/telogen ratio) improved progressively, increasing by 43% at week 4, 55% at week 8, and 73% at week 12 (*p* < 0.001; Figure 3c). The sensitivity analysis in the PP population (excluding outliers) yielded comparable results.

### 3.2 Hair Loss (Shampoo Test)

**FIGURE 4:**
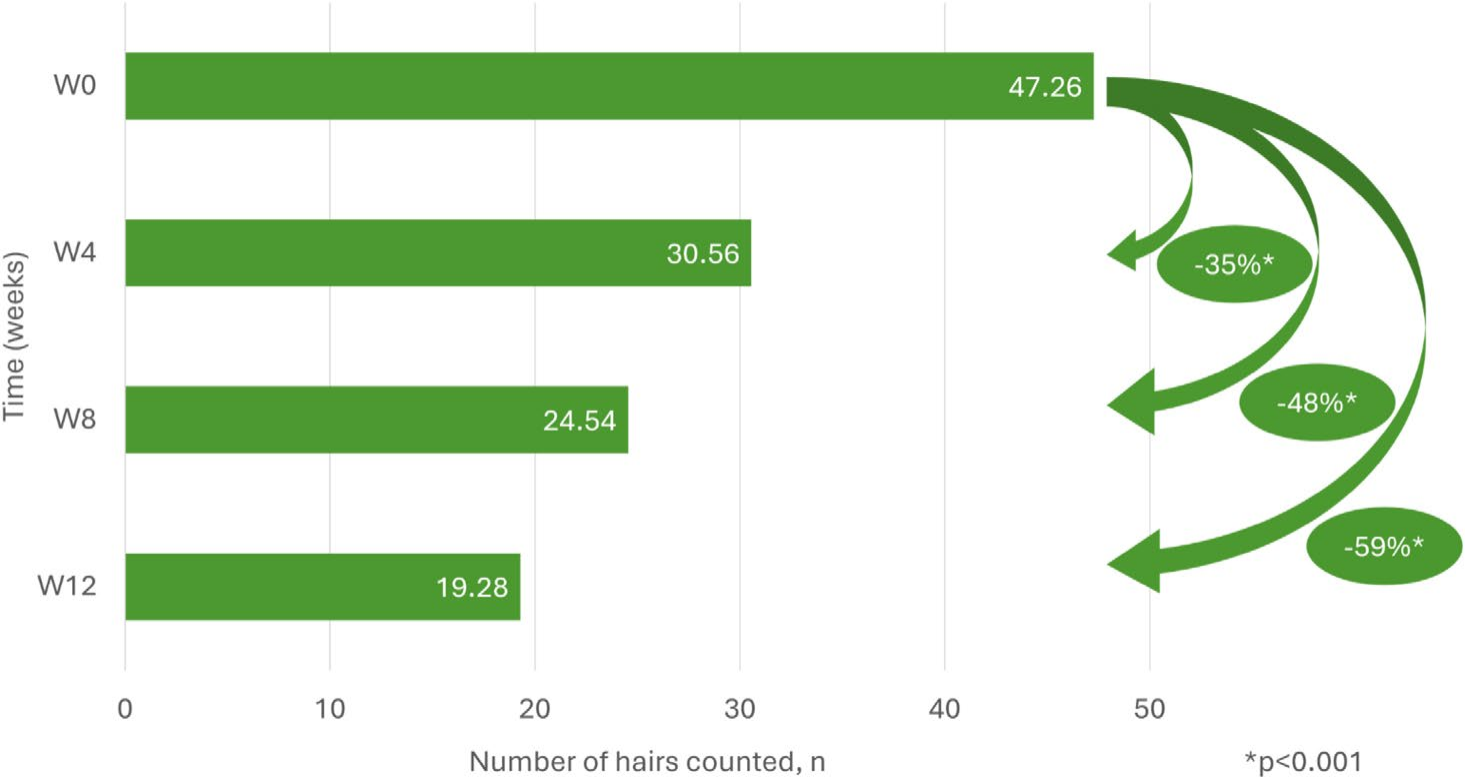
Change in hair loss during shampooing from baseline to week 12.

The mean number of hairs that were shed during shampooing decreased by 35% at week 4, 48% at week 8, and 59% at week 12 compared with baseline (*p* < 0.001) (Figure 4, Table S5). A similar decrease was noted in the sensitivity analysis.

### 3.3 Hair Appearance (Hairdresser Evaluation)

**FIGURE 5:**
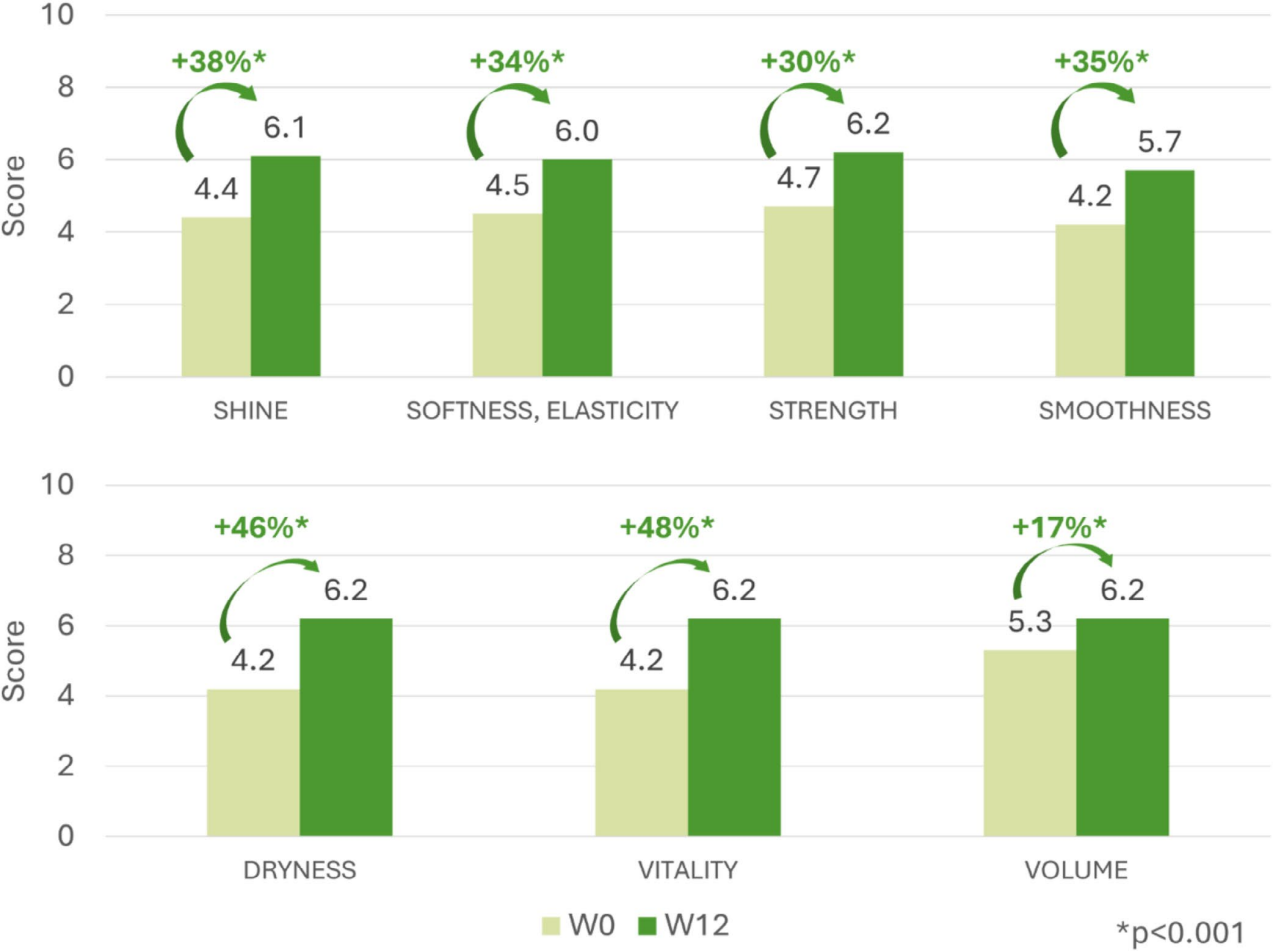
Change in hair appearance (hairdresser‐rated) from baseline to week 12.

Hairdresser‐assessed scores for shine, softness/elasticity, strength, smoothness, dryness, vitality, and volume all showed improvement from baseline to week 12 (Figure 5, Table S6), but no increase in hair thickness was observed. Significant improvements were noted at weeks 4, 8, and 12 for hair dryness (*p* < 0.001) and vitality (*p* ≤ 0.004), weeks 8 and 12 for shine, softness/elasticity, smoothness (*p* < 0.001), and strength (*p* < 0.001), and week 12 for hair volume (*p* < 0.001), with similar results in the sensitivity analyses.

### 3.4 Subject‐Reported Outcomes

**FIGURE 6:**
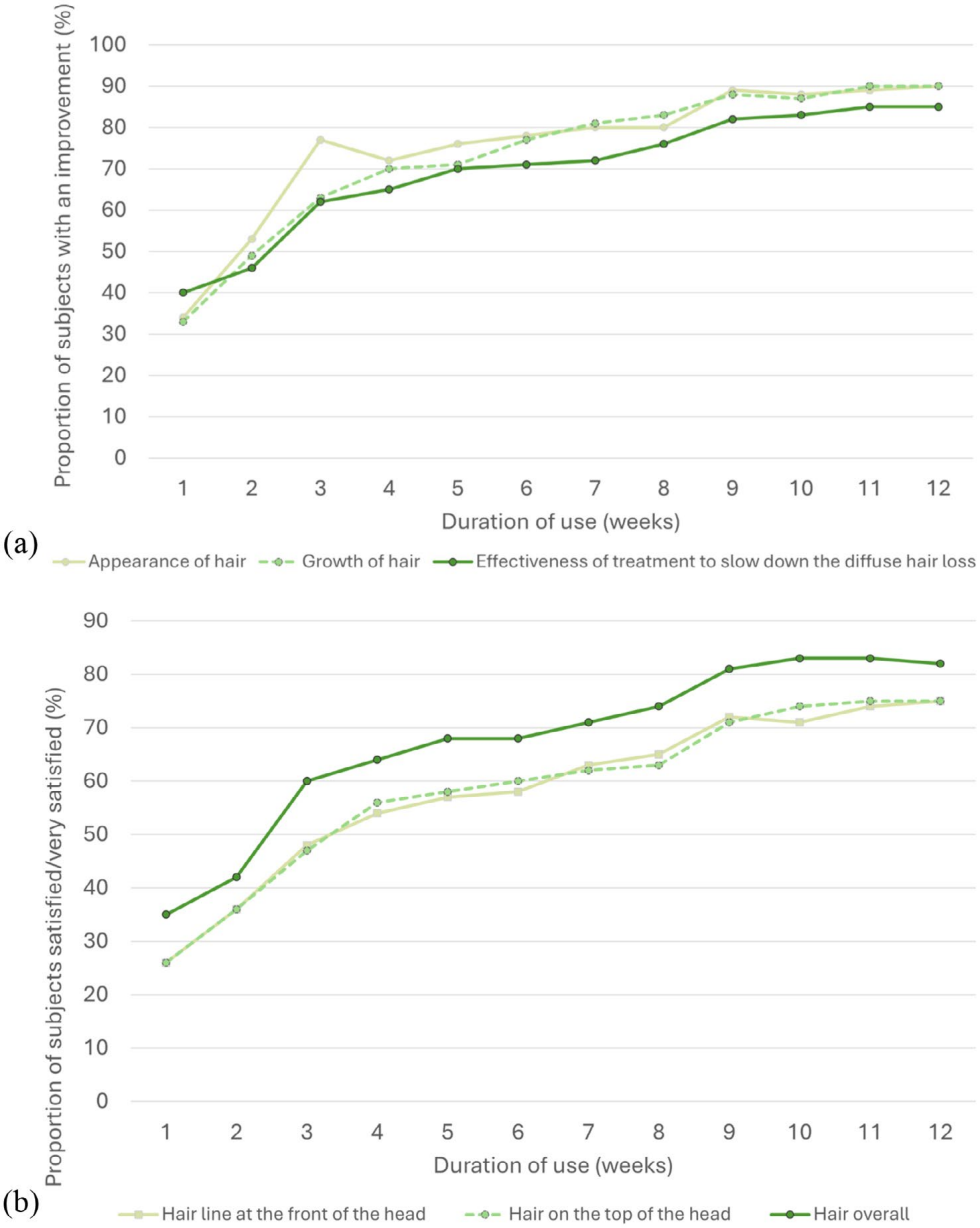
Change in (a) hair appearance (subject‐reported) and (b) patient satisfaction with their hair from weeks 1 to 12.

Weekly questionnaires completed by subjects indicated progressive improvement from week 2 in their perception of hair appearance, growth, and slowing of hair loss over the 12‐week period, with consistent upward trends for all satisfaction with hair scores (Figure 6, Table S7). At week 12, around 90% of subjects experienced a better hair appearance and increased hair growth, 85% rated the product as effective at slowing their hair loss, while 82% overall were satisfied with their hair overall.

### 3.5 Safety

The product was generally well tolerated. Overall, 66 subjects reported 139 treatment‐emergent AEs, of which the majority were mild (90.6%). Of these, 18.7% were headache, 13% were gastrointestinal events, and 7.9% were nasopharyngitis symptoms (considered by investigators to be unrelated to product use). Of the total AEs, 97 (69.8%) were considered by investigators to be related to the product; headache was the most frequent event, for which a causal relationship could not be ruled out with certainty.

With respect to local scalp tolerability, 31 subjects experienced at least one physical or functional sign on the scalp (reported by either the physician or the subject), which were mostly mild and transient. Events deemed clinically relevant (i.e., longer duration and/or greater intensity) occurred in 10 subjects (8.9% of the FAS population) and included greasy scalp (four subjects), dandruff (three), erythema (two), and pruritus (one). Conditions such as greasy scalp and dandruff were considered potentially influenced by the hormonal cycle. Only one serious AE (drug intoxication) was reported and was considered not related to the food supplement; the subject recovered without sequelae. No subject permanently discontinued treatment because of an AE.

## 4 Discussion

Hair appearance and beauty play an important role in self‐image and social perception, and changes such as thinning or loss can significantly affect psychological well‐being [2, 7, 8]. Consequently, interventions that support hair health and address diffuse hair loss are of considerable clinical and personal interest.

Our exploratory study in healthy individuals with non‐illness‐related, light diffuse hair loss found that use of a food supplement for 12 weeks significantly improved objective measures of hair growth and was well tolerated. We observed a progressive increase in total hair density, an improved hair growth coefficient (anagen/telogen ratio), and reduced hair loss. In addition, hairdresser‐rated assessments showed significant improvements in hair quality and appearance after 8 weeks, including shinier, softer, stronger, and smoother hair that was less dry and had more vitality. More than 80% of subjects were satisfied with treatment by the end of the study, with subjective improvements in hair appearance, growth, and hair loss reported from as early as week 2 and maintained over the 12‐week period.

The hair growth coefficient reflects the balance between actively growing (anagen) and resting (telogen) hairs on the scalp. In a healthy individual, around 85% of hairs are in the anagen phase and 15% in telogen [5]. A decrease in this ratio means that a smaller proportion of hairs are growing, which can lead to hair thinning and loss [19]. In our study, the anagen/telogen ratio improved significantly from 7.0 at baseline to 12.1 at 12 weeks. Similarly, the average number of hairs lost during the shampoo test decreased from 47 at baseline to 19 at week 12.

Other studies have also demonstrated beneficial effects of the individual ingredients or their combinations. For example, millet extract, L‐cystine, and calcium pantothenate (vitamin B5) used together for 6 months increased the proportion of anagen hairs from abnormal (< 80%) to normal levels (> 85%) in women with androgenetic alopecia—an effect that was significant compared with the placebo group [20]. In a non‐interventional study in people with androgenetic alopecia, 90 days of supplementation improved hair density, hair loss, and hair thickness (subject evaluation), with 75% of participants satisfied at study end [21]. In a double‐blind trial of men and women with diffuse hair loss and structural hair damage, supplementation with L‐cystine and calcium pantothenate for 4 months significantly improved hair swelling as a parameter for hair quality and increased the anagen rate in 75% of patients compared with 58% in the placebo group [22]. Together, these findings support the potential value of combining these ingredients for the management of diffuse hair loss.

Our study design has several advantages, but also limitations. Conducting the study across several centers increases the diversity of participants and settings, supporting the broader relevance of the findings. However, our statistical model did not evaluate potential center differences that may have influenced our findings; thus, we cannot fully exclude site‐to‐site variability. The use of healthy volunteers provides a controlled environment with fewer confounding factors, but this may limit the direct applicability of the results to patients with other types of hair loss whose hair cycle dynamics and scalp physiology may differ. While an open‐label design facilitates feasibility and reflects real‐world use, it also introduces the possibility of expectation bias, particularly for outcomes that rely on subjective assessment. Of particular importance is that during the winter months, spontaneous normalization of seasonal hair shedding may occur. Although the magnitude and progressive nature of the changes that we observed over 12 weeks suggest a potential treatment effect, in the absence of a placebo‐controlled comparator a contribution of natural seasonal variation cannot be excluded. This limitation will need to be addressed in future studies. Furthermore, the assessment of hair appearance, beauty, and volume by the hairdresser was subjective. However, the hairdressers are experts in their field and all used a standardized, clearly defined scale to ensure that they correctly understood the values assigned. Finally, the absence of a comparator arm restricts interpretation of the magnitude of effect, and inter‐site variability may still affect consistency despite standardization procedures.

In summary, this exploratory study suggests that the product containing millet extract, pantothenic acid, L‐cystine, and biotin may be effective and well tolerated for non‐illness‐related, light diffuse hair loss, thinning hair, and hair appearance and beauty—although controlled clinical trials are required to confirm these findings.

## Author Contributions

Writing – review and editing, Ehrhardt Proksch and Sandra Bartylla; visualization, Ehrhardt Proksch and Sandra Bartylla; both authors have read and agreed to the published version of the manuscript.

## Funding

This work was supported by Bayer Consumer Care (Basel, Switzerland).

## Ethics Statement

Institutional Review Board Statement: This exploratory food study did not require Ethics Committee Approval or the Competent Authority Authorization from countries involved (France, Poland). The study was conducted in accordance with the Declaration of Helsinki and local French and Polish regulations.

## Consent

Informed consent was obtained from all subjects involved in the study.

## Conflicts of Interest

Prof. Dr. Ehrhardt Proksch has previously received honoraria for speaking at symposia. Sandra Bartylla is an employee of Steigerwald Arzeimittelwerk GmbH, a subsidiary of Bayer Vital GmbH. The authors report no other possible conflicts of interest in this work. The sponsor, Bayer Consumer Care, was involved in the design of the study; in the writing of the manuscript; and in the decision to publish.

## Supporting information


**Table S1:** Inclusion and exclusion criteria.
**Table S2:** Study variables.
**Table S3:** Schedule of assessments.
**Table S4:** Hair growth outcomes (phototrichogram), full analysis set.
**Table S5:** Hair loss during shampoo test, full analysis set.
**Table S6:** Hairdresser evaluation scores for hair appearance, full analysis set.
**Table S7:** Subject‐reported outcomes for perceptions of hair appearance, growth, and slowing of hair loss (weekly questionnaire), full analysis set.

## Data Availability

The data that support the findings of this study are available from the corresponding author upon reasonable request.
