# Supplementary material for: Effectiveness, Tolerability, and Acceptance of a Food Supplement in the Management of Non‐Illness‐Related Hair Loss and Thinning
Source: J Cosmet Dermatol. 2026 May 17;25:e70852. doi: 10.1111/jocd.70852 (PMC13181224; doi:10.1111/jocd.70852)
Supplement: Supplementary file 1 — Table S1: Inclusion and exclusion criteria. Table S2: Study variables. Table S3: Schedule of assessments. Table S4: Hair growth outcomes (phototrichogram), full analysis set. Table S5: Hair loss during shampoo test, full analysis set. Table S6: Hairdresser evaluation scores for hair appearance, full analysis set. Table S7: Subject‐reported outcomes for perceptions of hair appearance, growth, and slowing of hair loss (weekly questionnaire), full analysis set. [file JOCD-25-e70852-s001.pdf]

Effectiveness, tolerability, and acceptance of a food supplement in the management of non-illness hair loss and thinning

Supplementary Material

Table S1. Inclusion and exclusion criteria. 2
Table S2. Study variables. 4
Table S3. Schedule of assessments....................5
Table S4. Hair growth outcomes (phototrichogram), full analysis set. 6
Table S5. Hair loss during shampoo test, full analysis set. 7
Table S6. Hairdresser evaluation scores for hair appearance, full analysis set. 8
Table S7. Subject-reported outcomes for perceptions of hair appearance, growth, and slowing of hair loss (weekly questionnaire), full analysis set. 9

Table S1. Inclusion and exclusion criteria.

Inclusion criteria	Exclusion criteria
• Healthy male or female.<br>• Caucasian subjects.<br>• Aged between 18 and 45 years, inclusive.<br>• Informed, written consent provided.<br>• Subject willing to adhere to the protocol and study procedures.<br>• Female subjects of childbearing potential must have a) had a negative pregnancy test at inclusion, and b) used a reliable form of contraception for at least 1 month prior to inclusion (3 months on oral contraceptives). Oral contraceptives, intrauterine devices (IUD), mechanical barrier method or abstinence were considered reliable methods of contraception.<br>• Subject had health insurance.<br>• Skin type I to IV according to Fitzpatrick.<br>• Subject confirmed to occasionally suffer from mild seasonal hair loss (in the previous spring or autumn).<br>• Proportion of hair in the telogen phase <20% for men and <15% for women, assessed in a previous study conducted by Dermscan a maximum 8 weeks before inclusion.<br>• At inclusion, increase in the percentage of hair in the telogen phase regarding the last evaluation done by Dermscan a maximum 8 weeks before inclusion.<br>• Proportion of hair in telogen phase >12% for subjects not previously pre-included by Dermscan (proportion of subjects will be presented in the statistical analysis).<br>• Weakened, devitalized hair with a loss of vitality such as hair dryness and reduced hair volume.<br>• Minimum of 6 cm of hair length and willingness to keep this hair length throughout study duration.<br>• Agreement to have a zone (1 cm^2) shaved on the scalp for phototrichogram purposes.<br>• Subject with history of hair dying must avoid any hair dying 3 weeks prior to Baseline and throughout the study.<br>• Hair of chestnut color or brown hair easily visible on photographs.<br>• Agreement to refrain from the use of any medical or cosmetic products that would influence hair loss/hair growth or hair beauty 30 days before Baseline and throughout the study.	• Subject with frizzy and very curly hair.<br>• For females: pregnant or nursing women or women planning pregnancy during the study.<br>• Use of hair gel, hairspray, or combing products on the days of measurement.<br>• Presence of white hair on the studied zone.<br>• History and current skin allergy, food allergy and or intolerance to food (including intolerance to gluten).<br>• History of skin allergy.<br>• Allergy to any of the ingredients and excipients of the test product.<br>• Subject with gastro-intestinal disease/disorders that could interfere with the absorption of food and food supplements.<br>• History of atopic dermatitis.<br>• Subject planning to modify their hair washing and hair care products and hair care procedures (shampoo, conditioner, etc., frequency of hair care).<br>• Subject planning to modify their hair style habits.<br>• Any systemic disorder or abnormal scalp condition (e.g., seborrheic dermatitis, alopecia, atopic dermatitis, dandruff, eczema, or psoriasis).<br>• Need for medical treatment which, in the opinion of the Investigator, could compromise the safety of the subject or affect the outcome of the study.<br>• History of medical/surgical events that, in the opinion of the Investigator, could affect the outcome of the study.<br>• Any topical or systemic medical treatment affecting hair growth or hair loss during the 6 months preceding the preinclusion (e.g., hormone replacement therapy, prostaglandins, rubefacients agents, all vasodilators, anti-androgens, all local hormonal treatment).<br>• Subject having undergone a surgical intervention under general anesthesia in the month before the beginning of the study.<br>• Subject under medical treatment affecting their immune response (e.g. corticosteroids, antihistamines, immunomodulators or any other medication/remedies that could have an impact on immune response).<br>• Exposure to excessive or intensive ultraviolet (UV) light (natural or artificial) on the investigational areas prior to the study.

	• Enrollment in another clinical trial 90 days prior to Baseline or another cosmetic trial 30 days prior to Baseline and throughout the whole study.<br>• Subjects belonging to the staff of the center or related to them, or employees and family members of the Sponsor and the CRO of the sponsor.<br>• Vaccination 3 weeks prior to Baseline or planning to get vaccinated during the study.<br>• Planned exposed to excessive sunlight during the study.<br>• Excessive use of alcohol or tobacco.<br>• Drug and alcohol addicts.

Table S2. Study variables.

Variable	Procedure	Timepoint (weeks)
**Hair density**, proportion and density of anagen and telogen hair, anagen/telogen ratio (hair growth coefficient)	**Phototrichogram** (digital images obtained using Nikon D7100 camera + Canfield Epiflash® with contact lens; image analysis using Photoshop CS6 extended® plugin). A 1 cm^2 test area was defined on the scalp of each subject using anatomical markers. The area was shaved at baseline and again at Weeks 4, 8, and 12, with follow-up photographs taken 2 days later without shaving. Within a defined 0.7 cm^2 region, the total number of hairs was manually counted and classified as anagen, telogen, or undetermined. Variables derived included hair density (hairs/cm^2), proportion and density of anagen hairs (A), proportion and density of telogen hairs (T), and the hair growth coefficient (anagen/telogen ratio, A/T).	0, 4, 8, 12
**Hair loss** (shed hairs)	Evaluated using a standardized hair washing procedure performed by a professional hairdresser. Lost hairs were collected via a filter in the sink and counted manually by the hairdresser.	0, 4, 8, 12
**Hair appearance** (hairdresser evaluation)	A professional hairdresser assessed eight parameters of hair quality on a 10-point scale:<br>• Shine (1=dull hair, 10=shiny hair)<br>• Softness/elasticity (1=rough & stiff hair, 10=soft & elastic or pliant hair)<br>• Strength (1=breakable hair, 10=strong hair)<br>• Smoothness (1=frizzy (‘fried-looking’) hair, 10=smooth, healthy-looking hair)<br>• Thickness (1=fine (vellous-like) hair, 10=thick (terminal) hair)<br>• Dryness (1=dry hair, 10=moisturized hair)<br>• Vitality (1=limp & lifeless hair, 10=healthy hair & full of vitality)<br>• Volume (1=lack of volume, 10=lots of volume)<br>Higher scores indicated better hair quality.	0, 4, 8, 12
**Acceptance** (subject evaluation)	Subjects completed weekly questionnaires throughout the study, recording changes in hair status compared with baseline. Items covered hair appearance, perceived growth, slowing of hair loss, satisfaction with frontal hairline, and overall satisfaction with hair status.	Weekly (0–12)
**Safety**	Assessed by a physician at each visit. Adverse events and serious adverse events were collected, with documentation of onset, intensity (mild, moderate, severe), relation to the investigational product, and outcome. Withdrawals due to AEs or intercurrent illness were fully documented. Compliance with product use and completion of subject diaries were checked at each visit.	Each visit (0, 4, 8, 12)

Table S3. Schedule of assessments.

Week	Key assessments performed
0	Baseline: demographics, medical history, pregnancy test (if applicable), inclusion/exclusion criteria, phototrichogram, standardized hair wash and hair count, hairdresser assessment (appearance/beauty/volume), profile photographs, clinical exam, AE collection, product dispensation, subject questionnaires
4	Phototrichogram, hair wash and hair count, hairdresser assessment, profile photographs, compliance check, AE collection, product dispensation, clinical exam, subject questionnaires
8	Phototrichogram, hair wash and hair count, hairdresser assessment, profile photographs, compliance check, AE collection, product dispensation, clinical exam, subject questionnaires
12	Final: phototrichogram, hair wash and hair count, hairdresser assessment, profile photographs, compliance check, AE collection, clinical exam, subject questionnaires, pregnancy test (if applicable), product return

Table S4. Hair growth outcomes (phototrichogram), full analysis set.

Timepoint	Density (hairs/cm^2)			Proportion in hair phase (%)		Growth coefficient
	Total	Anagen	Telogen	Anagen	Telogen	(anagen/telogen)
Baseline	223.8±68.1	190.8±59.0	33.0±15.6	85.3±5.4	14.7±5.4	7.0±3.9
Week 4	226.7±67.2	199.0±59.0**	27.7±16.0**	88.0±5.5**	12.0±5.5**	10.1±9.6**
Week 8	227.7±65.6*	201.2±56.7**	26.5±18.7**	88.6±6.1**	11.4±6.1**	10.9±8.2**
Week 12	228.9±68.9*	203.8±60.9**	25.1±18.7**	89.3±6.2**	10.7±6.2**	12.1±8.9**

Presented as mean ± standard deviation. *p≤0.005 vs. baseline; **p<0.001 vs. baseline.

Table S5. Hair loss during shampoo test, full analysis set.

Timepoint	Lost hairs (n), mean ± SD	Percent change from baseline (%)
Baseline	47.3±52.5	
Week 4	30.6±25.5*	-35
Week 8	24.5±25.0*	-48
Week 12	19.3±19.7*	-59

Presented as mean ± standard deviation. *p<0.001 vs. baseline.

Table S6. Hairdresser evaluation scores for hair appearance, full analysis set.

Timepoint	Hair appearance scores [a]							
	**Shine**	**Softness/ elasticity**	**Strength**	**Smoothness**	**Thickness**	**Dryness**	**Vitality**	**Volume**
Baseline	4.4±1.4	4.5±1.3	4.7±1.4	4.2±1.2	5.3±1.8	4.2±1.5	4.2±1.5	5.3±1.8
Week 4	4.5±1.4	4.6±1.4	4.5±1.4	4.5±1.6	4.9±1.7**	4.7±1.7***	4.7±1.7***	5.3±1.5
Week 8	5.0±1.2*	5.1±1.18*	5.3±1.5*	5.1±1.3*	5.0±1.4	5.3±1.6*	5.4±1.6*	5.3±1.5
Week 12	6.1±1.6*	6.0±1.6*	6.2±1.6*	5.7±1.8*	5.7±1.7	6.2±1.8*	6.2±1.8*	6.2±1.8*
Percent change from baseline (%)								
Week 8	+13	+13	+12	+20	—	+24	+26	—
Week 12	+38	+34	+30	+35	—	+46	+48	+17

Presented as mean ± standard deviation. *p<0.001 vs. baseline; **p=0.04 vs. baseline; ***p<0.005 vs. baseline. [a] Scored using scales of 1-10 (see Table S2), where higher scores indicate better hair quality.

Table S7. Subject-reported outcomes for perceptions of hair appearance, growth, and slowing of hair loss (weekly questionnaire), full analysis set.

Timepoint	Appearance [a]	Growth [b]	Slowing of hair loss [c]	Satisfaction with treatment [d]		
				Hairline (front)	Hair (top)	Hair overall
Week 4 [e]	78 (71.6)	76 (69.7)	71 (65.1)	59 (54.1)	59 (56.2) [g]	67 (63.8) [g]
Week 8 [f]	88 (80.0)	91 (82.7)	84 (76.4)	71 (64.5)	69 (62.7)	81 (73.6)
Week 12 [e]	98 (89.9)	98 (89.9)	93 (85.3)	82 (75.2)	82 (75.2)	89 (81.7)

Presented as number of subjects, n (%).[a] A little better to a lot better; [b] slightly increased to greatly increased; [c] somewhat effective to very effective; [d] satisfied to very satisfied; [e] n=109, unless indicated otherwise; [f] n=110; [g] n=105.
